# On the Inside

**DOI:** 10.1093/plphys/kiab042

**Published:** 2021-04-21

**Authors:** Peter V Minorsky

**Affiliations:** School of Health and Natural Sciences, Mercy College, Dobbs Ferry, New York, USA

## Seawater exposure and the decline of Sitka spruce trees

Sea level rise in response to global climate change increases the extent and duration of coastal inundation and seawater exposure, leading to reduced tree growth, failure in regeneration, and forest mortality events. These vulnerabilities in coastal forests have profound impacts on coastal biogeochemical cycling by altering vegetation composition, water/carbon fluxes, and sediment transport. Although forest mortality events (e.g., “ghost forests”) have been documented in many coastal regions, little is known about the mechanisms of forest die-off under seawater exposure. Despite the crucial role of nonstructural carbohydrate (NSC) reserves in tree survival, their dynamics in the process of death under seawater exposure are unknown. Zhang et al. (pp. 1682–1696) have monitored progressive tree mortality and associated NSC storage in Sitka-spruce (*Picea sitchensis*) trees dying under ecosystem-scale increases in seawater exposure in western Washington, DC, USA. Trees exposed to seawater experienced declining crown foliage during the sampling period. Individuals with a lower percentage of live foliated crown (PLFC) died faster. Tree PLFC was strongly correlated with subsurface salinity and needle ion contents. Total NSC concentrations in trees declined remarkably with crown decline and reached extremely low levels at tree death. Starch in all tissues was almost completely consumed while sugars remained at a homeostatic level in foliage. The decreasing NSC with the approach of death highlights the importance of carbon storage as an indicator of tree mortality risks under seawater exposure.

## Brassinosteroids suppress climacteric fruit ripening

The onset of ripening in climacteric fruit requires the increased biosynthesis of the gaseous hormone ethylene. This physiological process has practical applications in that the reduction of ethylene in climacteric fruit leads to a slower rate of fruit softening and longer shelf-lives. Although ethylene is the key hormone involved in climacteric fruit ripening, there is evidence that at least four other plant hormones, including abscisic acid, indole-3-acetic acid, jasmonate, and brassinosteroid (BR) are involved in regulating ethylene levels during climacteric fruit ripening. Among these four hormones, only the mechanisms by which BRs affect ethylene biosynthesis during ripening remain largely unknown. Ji et al. (pp. 1875–1893) now report that exogenous BR treatment suppressed ethylene production and delayed fruit ripening in pear (*Pyrus ussuriensis*), whereas treatment with a BR biosynthesis inhibitor promoted ethylene production and accelerated fruit ripening. These results suggest that BR may act as a ripening suppressor. The expression of the transcription factor BRASSINAZOLE-RESISTANT (PuBZR) was enhanced by BR treatment during pear fruit ripening. PuBZR1 interacted with PuACO1, which converts 1-aminocyclopropane-1-carboxylic acid (ACC) to ethylene and suppresses its activity. BR-activated PuBZR1 bound to the promoters of two genes encoding ACC synthase, and directly suppressed their transcription. Ethylene production and expression profiles of corresponding apple (*Malus domestica*) homologs showed similar changes. Together, these results suggest that BR-activated BZR1 suppresses ACO1 activity and the expression of *ACC synthase* genes, thereby reducing ethylene production and suppressing fruit ripening. This likely represents a conserved mechanism by which BR suppresses ethylene biosynthesis during climacteric fruit ripening.

## Cell-type-specific aquaporin dynamics

Plasma membrane intrinsic proteins (PIPs) are aquaporins that facilitate the passive diffusion of water and small neutral solutes across membranes. PIPs play a key role in facilitating the osmotic flows that underlie the stomatal movements of plants. Plant pathogens, including fungi and bacteria, induce stomatal closure as part of a plant defense mechanism aimed at limiting the entry of the pathogen into leaves. For example, the flagellin-derived peptide flg22 is perceived by the receptor FLAGELLIN SENSITIVE2, resulting in stomatal closure in wild-type Arabidopsis (*Arabidopsis thaliana*) plants. Following perception, several types of cation channels involved in stomatal closure as well as AtPIP2;1 are activated to facilitate the osmotic loss of water from guard cells, thereby leading to stomatal closure. In stomatal complexes, subsidiary cells surround guard cells and participate in rapid stomatal movements. Bulk water transfer also occurs in subsidiary cells during stomatal opening and closure. Although subsidiary cells are considered to function as important buffering machinery in the regulation of guard cell turgor and stomatal movement, whether the mechanism regulating hyperpolarization and turgor pressure in these cells during pathogen-triggered stomatal closure is the same as that in guard cells is currently unclear. Cui et al. (pp. 1666–1681) have examined in vivo the aquaporin dynamics that occur during the stomatal immune response to the bacterial flagellin-derived peptide flg22. They report that the Arabidopsis aquaporin mutant *pip2;1* shows defects in the flg22-induced stomatal response. The authors’ results also reveal that the movement dynamics and dwell times of GFP-AtPIP2;1 in guard cells and subsidiary cells exhibit cell-type-specific dependencies, and that the cytoskeleton, rather than the cell wall, is the major factor regulating AtPIP2;1 dynamics. These findings provide valuable information about the cell-type-specific mechanisms regulating water transport within the subsidiary and guard cells during plant defense responses.

## UV-dependent photoprotection in Chlamydomonas

In photosynthetic organisms, the nonphotochemical quenching (NPQ) of excess light energy prevents the generation of reactive oxygen species and maintains efficient photosynthesis under stressfully high light conditions. In the unicellular green alga *Chlamydomonas reinhardtii*, NPQ is activated as a photoprotective mechanism through wavelength-specific light signaling pathways mediated by the phototropin (blue light) and ultra-violet light (UV) photoreceptors, but the biological significance of photoprotection activation by light with different qualities remains poorly understood. Tokutsu et al. (pp. 1894–1902) demonstrate that NPQ-dependent photoprotection in *C. reinhardtii* is activated more rapidly by UV than by visible light and that the induction of gene expression and protein accumulation related to photoprotection is significantly faster and greater in magnitude under UV treatment compared to that under blue- or red-light treatment. Furthermore, the action spectrum of UV-dependent induction of photoprotective factors suggests that *C. reinhardtii* senses relatively long-wavelength UV (including UV-A/B), whereas the model dicot plant Arabidopsis preferentially senses relatively short-wavelength UV (mainly UV-B/C) for induction of photoprotective responses. The authors hypothesize that *C. reinhardtii* developed a UV response distinct from that of land plants.

## Interplay between autophagy and lipid metabolism

Under conditions of low energy availability, plants catabolize proteins, lipids, and chlorophylls instead of sugars to provide alternative substrates for the tricarboxylic acid cycle, thereby assuring the continued operation of the mitochondrial electron transport chain. Among the alternative substrates available for plant respiration, lipids comprise a significant carbon reserve that represents an important alternative source of energy. Plants store lipids in the form of triacylglycerol (TAG). In leaf mesophyll cells, TAG is deposited in specialized storage compartments, namely lipid droplets (LDs) in the cytosol or plastoglobuli (PG) inside chloroplasts. During nutrient deprivation, cellular lipid reserves are hydrolyzed into fatty acids, which are further catabolized during β-oxidation. Autophagy, one of the primary cellular catabolic processes, is involved in the degradation and recycling of cytoplasmic constituents into primary metabolites. The importance of autophagy in plants has been mostly associated with the recycling of proteins to amino acids under starvation conditions. In contrast, our understanding of the function of autophagy in the management of cell lipid reserves is fragmentary. To further investigate the significance of autophagy in lipid metabolism, Barros et al. (pp. 1542–1558) performed an extensive lipidomic characterization of Arabidopsis autophagy mutants (*atg*) subjected to dark-induced senescence conditions. Their results reveal an overall impact of autophagy deficiency on all lipid classes analyzed as well as the free fatty acid profile. The differential pattern of phospholipid levels and TAG accumulation was associated with changes in LD number and PG size. In addition, analysis of β-oxidation genes revealed an induction of lipid oxidation pathways in *atg* mutants. Collectively, these results demonstrate that autophagy is an essential component in the management of lipid reserves, affecting membrane lipid mobilization, the balance of LD and PG formation, and the regulation of β-oxidation pathways under dark-induced senescence.

## BR and the development of the pulvinus

Nyctinastic leaf movements occur widely in leguminous plants and are generated by a specialized motor organ, the pulvinus. Efforts have been made to understand the genetics underlying the development of pulvini. Mutants in which the pulvinus develops abnormally have been identified in *Medicago truncatula* (*petiolule-like pulvinus*/*plp*; *elongated petiolule1*/*elp1*) and a handful of other legume species. In these mutants, the pulvinus is replaced by a petiolule-like structure, the motor cells being replaced by elongated petiole-like epidermal cells. PLP/ELP1 is a LATERAL ORGAN BOUNDARIES domain (LBD) transcription factor, which is represented in the nonpulvinus forming species *A. thaliana* by a protein called LATERAL ORGAN BOUNDARIES (LOB). *PLP*/*ELP1*, as the putative ortholog of *LOB* in *M. truncatula*, has been shown to be required for the determination of the pulvinus. In Arabidopsis, *LOB* forms a feedback loop to orchestrate local BR accumulation during organ boundary formation by directly activating *PHYB ACTIVATION-TAGGED SUPPRESSOR1* (*BAS1*) transcription. If the pulvinus is considered to be the boundary between the leaflet and the petiole, then *LOB* and *PLP* define different boundaries. These considerations raise questions about whether the BR activity mediated by the *LOB*/*BAS1* module is involved in pulvinus development. Here, through an analysis of knockout mutants in barrel clover (*Medicago truncatula*), Kong et al. (pp. 1745–1763) reveal that neither altering BR content nor blocking BR signal perception affected pulvinus determination. However, BR homeostasis did influence nyctinastic leaf movement. Moreover, BR activity in the pulvinus is regulated by *BAS1*, which is directly activated by *PLP*/*ELP1*. A comparative analysis between *M. truncatula* and the nonpulvinus forming species *Arabidopsis* and tomato (*Solanum lycopersicum*) revealed that *PLP*/*ELP*1 may act as a factor that associates with unknown regulators in pulvinus determination in *M. truncatula*.

